# The Effect of Glucose Metabolism and Breastfeeding on the Intestinal Microbiota of Newborns of Women with Gestational Diabetes Mellitus

**DOI:** 10.3390/medicina58030413

**Published:** 2022-03-10

**Authors:** Miljana Z. Jovandaric, Svetlana J. Milenkovic, Ivana R. Babovic, Sandra Babic, Jelena Dotlic

**Affiliations:** 1 Department of Neonatology, Clinic for Gynecology and Obstetrics, University Clinical Center of Serbia, 11000 Belgrade, Serbia; ceca.milenkovic@yahoo.com; 2 Department of Gynecology and Obstretics, Clinic for Gynecology and Obstetrics, University Clinical Center of Serbia, 11000 Belgrade, Serbia; ivana.r.babovic@gmail.com (I.R.B.); sandradrmilic@yahoo.com (S.B.); drenadot@gmail.com (J.D.); 3 Medical Faculty, University of Belgrade, 11000 Belgrade, Serbia

**Keywords:** gestational diabetes mellitus, microbiota, breastfeeding, newborn

## Abstract

Gestational diabetes mellitus (GDM) is a pregnancy complication in which women without previously diagnosed diabetes develop chronic hyperglycemia during gestation. The diet and lifestyle of the mother during pregnancy as well as lactation have long-term effects on the child’s health and development. Detection of early risk markers of adult-age chronic diseases that begin during prenatal life and the application of complex nutritional interventions at the right time may reduce the risk of these diseases. Newborns adapt to the ectopic environment by developing intestinal immune homeostasis. Adequate initial colonization of bacteria is necessary for sufficient development of intestinal immunity. The environmental determinant of adequate colonization is breast milk. Although a developing newborn is capable of producing an immune response, the effector immune component requires bacterial stimulation. Breast milk stimulates the proliferation of a well-balanced and diverse microbiota, which initially influences the switch from an intrauterine TH2 predominant to a TH1/TH2 balanced response and the activation of T-regulatory cells by breast milk-stimulated specific organisms (Bifidobacteria, Lactobacillus, and Bacteroides). Breastfeeding in newborns of mothers with diabetes mellitus regulates the adequate immune response of the newborn and prevents diseases of the neonatal and postnatal period.

## 1. Introduction

### 1.1. Gestaional Diabetes Mellitus and Pregnancy

Gestational diabetes mellitus (GDM) is a pregnancy complication in which women without previously diagnosed diabetes develop chronic hyperglycemia during gestation. It is the most common medical complication and metabolic disorder of pregnancy. Prevalence may vary from 1 to 14% among all pregnancies [1,2]. There is evidence that microbial organisms within the gut—the “gut microbiome”—might contribute to metabolic diseases, including GDM. The gut microbiome can be influenced by early life events, such as preterm delivery and breastfeeding, and by events in later life, such as diet composition and antibiotic use. The gut microbiome has been consistently reported to differ between metabolically healthy and obese individuals, including during pregnancy [3]. GDM is a topic of great interest because it represents a major risk factor for adverse fetal maternal outcomes such as preeclampsia, preterm birth, fetal macrosomia, polyhydramnios, shoulder dystocia, Caesarean section, neonatal respiratory distress, neonatal hypoglycemia and perinatal mortality. An appropriate management of this disorder (adequate counselling, self-glucose monitoring, diet, physical activity, and eventually medication) is crucial for a favorable pregnancy outcome [4].

In women with GDM during pregnancy, there is a higher chance not only to develop type II diabetes mellitus during life, but also the risk for pancreatic cancer [5].

### 1.2. Glucose Metabolism with Gestational Diabetes Mellitus Pregnancy

Glucose metabolism in early pregnancy is characterized by hypoglycemia, hypoinsulinemia, ketogenesis, and fatty acid turnover. Between 24–28 weeks of gestation, the concentrations of placental and insulin-antagonistic factors increase, altering carbohydrate metabolism. This is interpreted as antagonism of the action of circulating insulin. Due to the reaction of the pancreas, insulin secretion increases. However, in some cases, the reserves of the pancreas can be reduced or depleted [6]. Due to the abnormal metabolism of carbohydrates, altered maternal glucose homeostasis develops, which is a condition referred to as DMG. The basis of the disorder is increased secretion of hPL (although growth hormone is reduced), as well as the effect of placental insulinases [7].

Insulin degradation on the maternal side of the placenta is unilateral, since insulin does not cross the placental barrier. The insulin resistance is of the post-receptor type and is hormonally conditioned. A decrease in the number of insulin receptors bound to peripheral target cells, along with a relative lack of circulating insulin, including post-receptor defects, leads to glucose intolerance and the development of gestational diabetes [8,9].

Changes in the mother’s glucose and insulin concentrations are followed by similar changes in the fetus. Maternal hyperglycemia leads to fetal hyperglycemia and hyperinsulinemia. The consequences of this excessive stimulation are reflected in the fetal pancreas in the form of islet hyperplasia with an increase in the number of beta cells and increased insulin values [10]. The importance of detecting gestational diabetes in the early stages of pregnancy is best illustrated by the data reporting a significant percentage of morbidity and mortality in newborns, in mothers in whom this disorder is undiagnosed and untreated, and in those when it is discovered late in pregnancy [11]. Fetal adiposity, which is also found in well-regulated diabetes, is explained by an increase in free fatty acids in the placenta, with increases the transfer to the fetus. The passage of amino acids through the placenta regulates insulin (hyperinsulinemia increases the transfer), and the fetal pancreas responds to an increase in the concentration of amino acids and glucose by producing insulin [12]. This is one of the important reasons for the occurrence of fetal macrosomia. It was found that the concentration of amino acids is higher in the fetal circulation than in the maternal circulation, while fetal glycemia is approximately 1.1–1.6 mmol/L lower than the maternal glycemia, i.e., 70–80% of maternal glycemia [13]. Medical nutritional therapy is the first-line approach in managing gestational diabetes mellitus (GDM). Diet is also a powerful modulator of the gut microbiota, whose impact on insulin resistance and the inflammatory response in the host are well known. Changes in the gut microbiota composition have been described in pregnancies either before the onset of GDM or after its diagnosis. The possible modulation of the gut microbiota by dietary interventions in pregnancy is a topic of emerging interest, in consideration of the potential effects on maternal and consequently neonatal health. To date, very few data from observational studies are available about the associations between diet and the gut microbiota in pregnancy complicated by GDM. In this review, we analyzed the available data and discussed the current knowledge about diet manipulation in order to shape the gut microbiota in pregnancy [14].

The priority of current studies is to discover mechanisms by which epigenetic modification prolongs the effects of environmental influences in early childhood and provides a long-lasting response to transient stimulus-modified gene expression and phenotype in adults [15].

Thus, nutrition in the pre- and postnatal period positively influences health in adulthood [16]. An adverse intrauterine environment in pregnancy complicated with diabetes has long-term consequences for the offspring because of the effects of epigenetics [17]. Optimal control of pregnant women’s glycemia can reduce the adverse consequences of pregnancy complicated with diabetes because the glucose level and perinatal outcome are a continuum [18]. Children of mothers with GDM are at greater risk of obesity, diabetes type 1 and 2, hypertension, lipid changes, and albuminuria in preadolescent age and in adulthood [19]. Mental and motor deficits as well as attention and behavioral disorders are much more common in the offspring of mothers with diabetes [20,21]. Many consequences of GDM during pregnancy can be prevented. Early breastfeeding can prevent metabolic complications in neonates because colostrum is rich in glucose, and hypoglycemia may be asymptomatic. GDM can lead to not only development of fetal macrosomia, neonatal hypoglycemia, jaundice, polycythemia, and hypocalcemia during the perinatal period, but also childhood obesity and metabolic syndrome in adulthood [22].

## 2. Gestational Diabetes Mellitus and Meconium Microbiota

The human body harbors trillions of microbial cells and they are indispensable for human health. The gut microbiota resides on the intestinal mucosal surfaces and participates in epithelial homeostasis, energy harvest, and immune development [23]. Colonization of the infant’s gut has drawn great interest, because it links to individual’s health and late-onset diseases [24]. However, factors that affect neonatal gut microbiota and metabolome in neonates of mothers with GDM has not been fully elucidated [25]. Microorganisms in meconium were the first colonizers of the newborn, which come from the mother’s skin, vagina, and gut. A wide variety of reports had demonstrated that microbiotain meconium could be affected by the delivery mode, perinatal antibiotics, and breastfeeding [26]. In the Ting Chen study, the relationship between the meconium microbiota, metabolome in neonates born to mothers with GDM was identified. A limited number of Taxa and Proteobacteria as the dominant phylum in the meconium of newborns of mothers with GDM was also found [27]. Decreased enteric microbiota richness is associated with increased insulin resistance markers and proinflammatory markers [28].

The abundances of the Rothia families and Clostridium sensustricto, which may contain opportunistic pathogens that might cause enteric infections and childhood metabolic disorders, were significantly increased in the GDM neonates. Decreased richness of the enteric microbiota has been associated with elevated insulin resistance and proinflammatory markers [29]. In addition, bacterial family changing was in the similar trend by delivery modes. It was indicated that variation of GDM-related bacteria was consistent. However, the consistency of changed bacteria in neonates might dramatically be altered within days based on feeding (breast/formula) enteric microbiota varies among different races, depending on the mother’s diet and climate [30].

## 3. Human Breast Milk

Breast milk contains many complex proteins, lipids and carbohydrates, the concentrations of which are altered dramatically over a single feeding and/or lactation to reflect the infant’s needs. Breast milk contains a myriad of biologically active components [31].

These molecules possess diverse roles, guiding the development of both the infant immune system and intestinal microbiota. It is believed that bacteria from the mother’s intestine may translocate to breast milk and dynamically transfer to the infant [32].

Such an interplay between the mother and her infant is key to establishing a healthy infant intestinal microbiome. These intestinal bacteria protect against many respiratory and diarrheal illnesses, but are subject to environmental stresses such as antibiotic use. Orchestrating the development of the microbiota are human milk oligosaccharides (HMOs), the synthesis of which is partially determined by the maternal genotype [33].

Human milk (HM) contains a large number of lipids that are essential for optimal growth and development. These lipids compose approximately 5% of the total milk profile and are packaged as milk fat globules. Milk fat globules are synthesized from triacylglycerol droplets within the endoplasmic reticulum of the mammary epithelium, and thus, the milk fat globule membrane (MFGM) is a tri-layer structure composed of many polar lipid species surrounding a nonpolar lipid core. The lipids of the MFGM make up approximately 2% of the HM lipidome and include phospholipids, such as phosphatidylinositol (PI), phosphatidylcholine (PC), phosphatidylethanolamine (PE), and phosphatidylserine (PS), and sphingolipids, such as SM and sphingomyelin ceramides (Cer) [34].

Breast milk fat is a major source of energy, providing over 40% of the total energy for infants during the first 6 months of life. Fat globules in breast milk consist of TAGs (98–99% of milk lipids) and small amounts of monoacylglycerols, diacylglycerols, and free fatty acids surrounded by milk fat membranes of different phospholipids [35]. Some of the indispensable lipid components include PUFAs and long-chain PUFAs (LC-PUFAs) of the n-6 and n-3 series and lipid-soluble vitamins. A recent study reported the complex interactions between fatty acids and protein profiles that were influenced by the lactation stage and gestational age. Regional dietary differences may also affect the breast milk composition, for example, increased consumption of n-3 PUFA (linoleic acid)-containing vegetable oil products in Western diets has led to an increase in the linoleic acid concentration from 50 to 90% of the PUFAs [36].

The influence of breast milk on the initial intestinal microbiota also prevents the expression of immune-mediated diseases (asthma, inflammatory bowel disease, and type 1 diabetes) later in life through a balanced initial immune response, underscoring the necessity of breastfeeding as the first source of nutrition [37].

Premature birth causes an interruption of lung development and maturation and simultaneously exposes the neonate to profound alterations of these systems. Abrupt interruption of placental nutrition, lung exposure to a highly oxidant environment, disruption of the nascent microbiome by invasive procedures, hospital microbiota, broad-spectrum antibiotics and many other factors lead to acute and chronic lung disease. Maternal diabetes mellitus and excessive fetal carbohydrate exposure alter surfactant synthesis in term and near-term infants. Animals born from induced GDM mothers show impaired lung development and maturation, with decreased expression of surfactant proteins B and C and their regulatory factor FOXA2 associated with inducible nitric oxide synthase induction and the generation of reactive oxygen species, which is a possible mechanism explaining the respiratory failure in infants of diabetic mothers [38].

In addition, antimicrobial factors such as lactoferrin, leukocytes, secretory IgA, complement factors, cytokines, lactoferrin and lysozyme play a fundamental immunomodulatory role. As recently discovered, human milk exosomes containing microRNA and other epigenetic factors interact with gene transcription and exert longstanding effects on gut homeostasis. Pasteurization, which is considered a standard for DBM out of concern for the transmission of infectious agents such as cytomegalovirus, human immunodeficiency virus and herpes simplex virus, inactivates many microcomponents and compromises its bactericidal and immunomodulatory properties [39].

GDM could alter the serum metabolome and is associated with the neonatal meconium microbiota and metabolome, highlighting the importance of breastfeeding on early life metabolism [40].

## 4. Conclusions

The diet and lifestyle of the mother during pregnancy as well as lactation have a long-term effect on the child’s health and development. Breastfeeding in newborns of mothers with diabetes mellitus prevents neonatal hypoglycemia, regulates the adequate immune response of the newborn and prevents diseases in the neonatal and postnatal period.

## Data Availability

All data are available in the archives (database) medline, PubMed.
